# Supplementary material for: Bouncing Dynamics of Impact Droplets on the Biomimetic Plane and Convex Superhydrophobic Surfaces with Dual-Level and Three-Level Structures
Source: Nanomaterials (Basel). 2019 Oct 25;9(11):1524. doi: 10.3390/nano9111524 (PMC6915665; doi:10.3390/nano9111524)
Supplement: Supplementary file 1 [file nanomaterials-09-01524-s001.pdf]

*Supplementary Materials*

# Bouncing Dynamics of Impact Droplets on the Biomimetic Plane and Convex Superhydrophobic Surfaces with Dual-Level and Three-Level Structures

**Zhongxu Lian[1], Jinkai Xu[1], Wanfei Ren[1], Zuobin Wang[1,2] and Huadong Yu[1,*]**

1 Ministry of Education Key Laboratory for Cross-Scale Micro and Nano Manufacturing, Changchun University of Science and Technology, Changchun, 130022, China; lianzhongxu@cust.edu.cn (Z.L.); xujinkai2000@163.com (J.X.); renwaifei@ccut.edu.cn (W.R.); wangzb@cust.edu.cn (Z.W.)

2 International Research Centre for Nano Handling and Manufacturing of China, Changchun University of Science and Technology, Changchun, 130022, China

* Correspondence: yuhuadong@cust.edu.cn

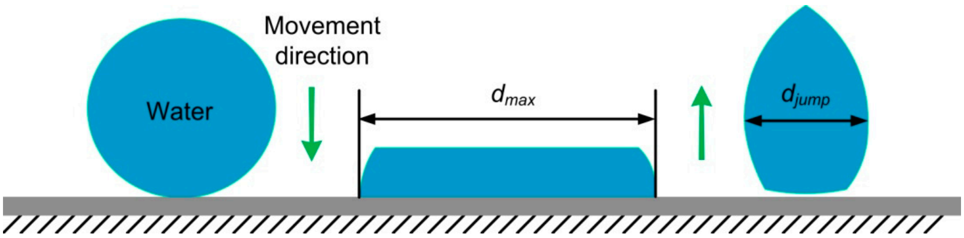


**Figure S1** Bounce diagram of water droplet on a superhydrophobic surface.

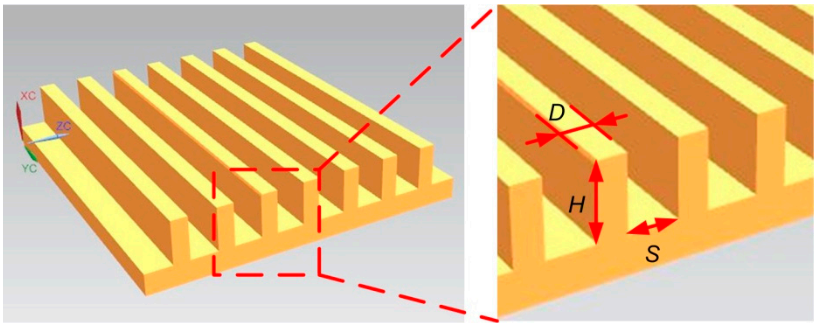


**Figure S2** Schematic diagram of three-level structured plane surface.
